# Parental behavior and newborn attachment in birds: life history traits and endocrine responses

**DOI:** 10.3389/fpsyg.2023.1183554

**Published:** 2023-08-03

**Authors:** Daniel Mota-Rojas, Míriam Marcet-Rius, Adriana Domínguez-Oliva, Jhon Buenhombre, Erika Alexandra Daza-Cardona, Karina Lezama-García, Adriana Olmos-Hernández, Antonio Verduzco-Mendoza, Cécile Bienboire-Frosini

**Affiliations:** ^1^Neurophysiology, Behavior and Animal Welfare Assessment, DPAA, Universidad Autónoma Metropolitana, Mexico City, Mexico; ^2^Department of Animal Behaviour and Welfare, Research Institute in Semiochemistry and Applied Ethology, Apt, France; ^3^Faculty of Veterinary Medicine, Antonio Nariño University, Bogotá, Colombia; ^4^Division of Biotechnology—Bioterio and Experimental Surgery, Instituto Nacional de Rehabilitación-Luis Guillermo Ibarra Ibarra, Mexico City, Mexico; ^5^Department of Molecular Biology and Chemical Communication, Research Institute in Semiochemistry and Applied Ethology, Apt, France

**Keywords:** imprinting, altricial, precocial, nesting, prolactin, corticosterone

## Abstract

In birds, parental care and attachment period differ widely depending on the species (altricial or precocial), developmental strategies, and life history traits. In most bird species, parental care can be provided by both female and male individuals and includes specific stages such as nesting, laying, and hatching. During said periods, a series of neuroendocrine responses are triggered to motivate parental care and attachment. These behaviors are vital for offspring survival, development, social bonding, intergenerational learning, reproductive success, and ultimately, the overall fitness and evolution of bird populations in a variety of environments. Thus, this review aims to describe and analyze the behavioral and endocrine systems of parental care and newborn attachment in birds during each stage of the post-hatching period.

## 1. Introduction

An intricate interplay between hormones and behavior controls parental care and attachment in both precocial and altricial birds (Vleck and Vleck, 2011; Smiley, 2019). From a developmental perspective, altricial birds are those where the hatchling is born without plumage, with closed eyes, limited locomotor activity, and stay in the nest for prolonged periods requiring parental care. In contrast, precocial birds newborns are born with down and contour feathers, with open eyes, and are able to leave the nest in shorter periods (Chen et al., 2019). The endocrine processes help regulate and coordinate parental care activities, including nesting, laying, incubating, hatching, feeding, protection and social bonding (Vleck and Vleck, 2011; Smiley, 2019). The specific hormonal profiles and interactions differ between precocial and altricial birds due to their distinct reproductive strategies and developmental needs (Cones and Crowley, 2020). However, in both cases, the endocrine system plays a crucial role in facilitating the expression of parental care and the formation of strong attachments between parents and their offspring.

Some birds, such as albatrosses or penguins, have a high level of parental investment and both parents take part in incubating eggs and caring for chicks while other birds, such as chickens and ducks, have a lower level of parental investment (the female will usually take on most of the caregiving responsibilities, such as incubating the eggs and caring for the chicks) (Ackerman et al., 2003; Zuk and Bailey, 2008; Morrison et al., 2016). Some other birds, such as the cuckoo, do not form bonds with their offspring and leave the care of their young to other birds (brood parasitism) (Mérő et al., 2023). However, generally, brooding and feeding the offspring is performed under biparental care in birds (Long et al., 2022), as observed in passerines (Vanadzina et al., 2023), doves (Farrar et al., 2022), and other non-passerine birds (Wagner et al., 2019). This means that the female and male are involved in different activities before the hatching period, to increase breeding success, save energy for both parents, and enhance their performance (Mock, 2022). As observed in mammals, for birds, parental care includes behavioral patterns from mate choice, nesting –site selection and construction according to the species– (Seltmann et al., 2017), egg size, time of incubation –from the onset of incubation to the first egg hatching–, and brood care, in order to maximize parental care. Notably, both the female and the male participate in the incubation of the eggs and develop behavioral strategies, such as turn-taking or synchronizing eating patterns (Kavelaars et al., 2019).

**Figure 1:**
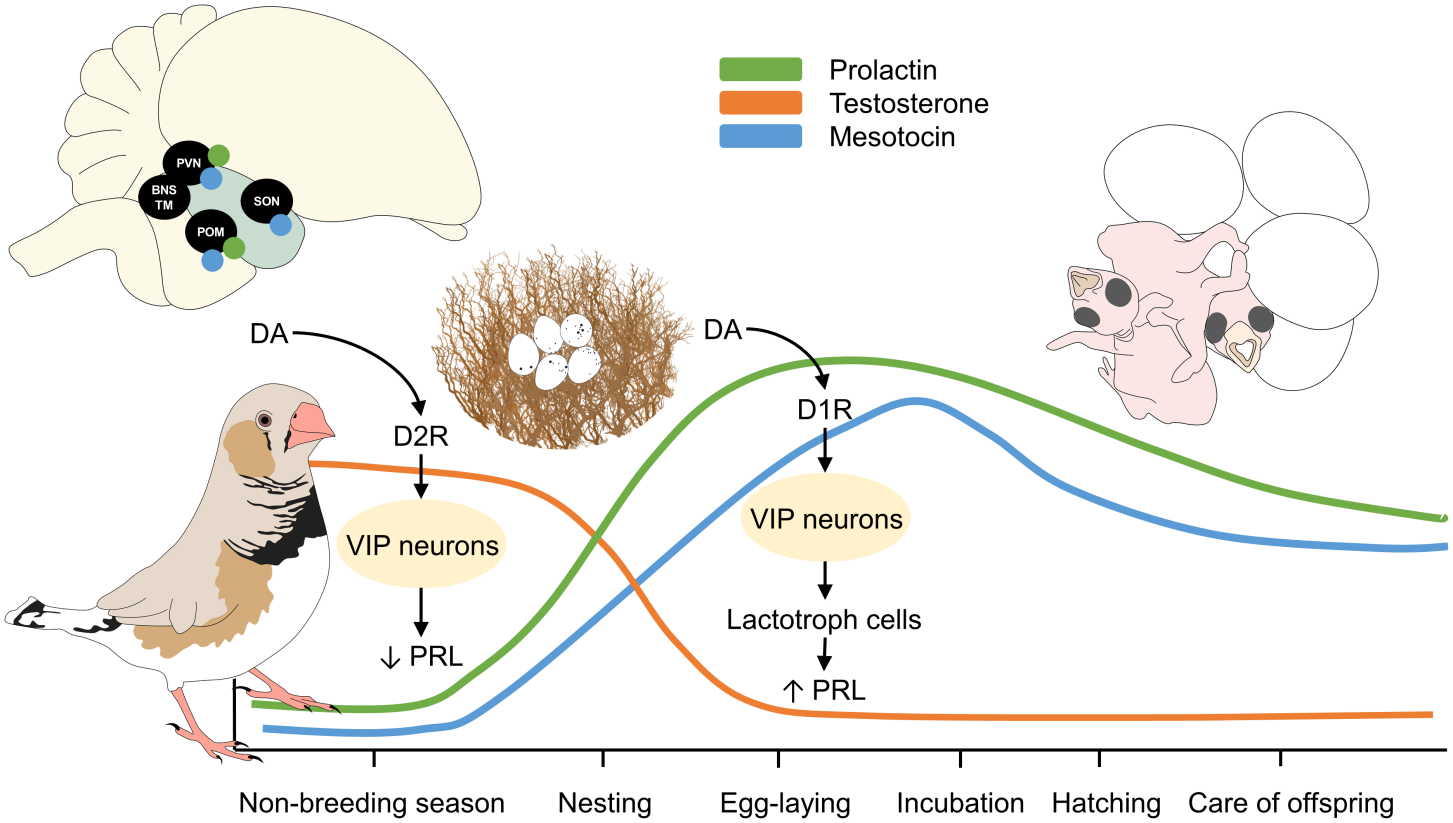
Phases of parental behavior in precocial and altricial birds. The depicted stages of parental behavior are characterized by the type of species (e.g., if birds build a nest or use pre-existing surfaces). As an example of a precocial bird, the hatchling of the domestic chick is born with functional sensory systems that facilitate its adaptation to the environment. In contrast, the African grey parrot –an example of altricial bird– has offspring with limited movement that requires longer rearing periods that is equivalent to increased parental care. The hormones involved are marked inside the orange rectangles, representing that its presence and increase modulates each stage. E2, estradiol; LH, luteinizing hormone; PRL, prolactin; T3, triiodothyronine.

In general, the breeding cycle of birds includes nesting, egg-laying, incubation, hatching, and care of the offspring during the post-hatching stage of development (Bates et al., 2022), periods that will be discussed in the present review and are schematized in Figure 1. These are highly influenced by neural and endocrine responses that generate behavioral patterns (Knerer and Atwood, 1966; Mainwaring et al., 2014). Parental behavior can also be influenced during the embryonic stage, as shown by Tuculescu and Griswold (1983) in hens, in which embryonic distress-calls promote hens’ maternal care by staying in the nest. In particular, the paraventricular nucleus (PVN), the medial preoptic area (referred to in birds as POM), and the supraoptic nucleus (SON) are some of the main cerebral structures involved in parental behavior (Lovell et al., 2008; Chokchaloemwong et al., 2013; Kuenzel et al., 2015; Aleksandrova, 2019; Smiley, 2019; Kelly and Adkins-Regan, 2020). When considering the endocrine biomarkers, high levels of prolactin (PRL) (Boos et al., 2007) and mesotocin (an analog of mammalian oxytocin), together with low levels of testosterone and corticosterone, dictate the differences observed between species and the establishment of the newborn attachment (Drobniak et al., 2022). Besides, estrogen is a hormone associated with nesting behavior, along with progesterone (P4). Nonetheless, as mentioned by Bluhm et al. (1983a), the endocrine responses are not solely linked to one hormone since luteinizing hormone (LH), PRL, estradiol, and P4 dictate the presence of parental behavior in birds, depending on the reproductive phase, and can be affected by external factors such as stress.

Several elements can alter the different phases such as species characteristics (e.g., altricial or precocial), environmental elements (e.g., temperature and the presence of predators), and nutritional quality, among other (Saino et al., 2001). That is why, for altricial animals, such as many birds and rodents, nests are especially important to protect offspring from heat loss and predators (Lynch and Possidente, 1978). For example, immediately after hatching, altricial offspring require constant nursing and feeding (Mock, 2022). The sensitive period for altricial birds is when they are in their early development period when they are still confined to the nest, and hence the importance of the success of the nest (Liebezeit and George, 2002). In contrast, although not all precocial hatchlings can fly within a few days, they can flee by running and are not highly vulnerable to temperature changes (Bertin et al., 2018). The temperature of the environment and within the nest is another element that could affect the bonding and nursing of offspring. Females can also compensate and adjust egg nutrients according to clutch size, growth rate, and survival (Bolton, 1991; Williams, 1994; Pelayo and Clark, 2003). Additionally, a highly relevant and interesting behavior known as brood parasitism (Cockburn, 2006; Lopes and de Bruijn, 2021) is considered another adaptative response of some avian species to enhance offspring fitness and ensure their survival.

One of the main challenges of achieving survival in birds is to develop nests that are suitable to help the young survive the climatic conditions and predators. Hence the importance of selecting a breeding site with a lower-than-average probability of reproductive failure (Emmering et al., 2018).

Filial imprinting is known as a type of social attachment and learning of young birds to the parents or an object (McCabe, 2019). Due to the complexity of the breeding cycle of birds and all the elements that can impair filial imprinting or nursing the offspring, this review aims to describe and analyze the neurobiological systems of parental behavior and newborn attachment in birds, according to the species: precocial and altricial birds. For each breeding stage, the behavioral traits will be explored and the neuroendocrine response and its association with certain hormones will be discussed to elucidate their impact in the post-hatching period.

## 2. Parental behavior in precocial species according to breeding stages

### 2.1. Nesting

Nest construction has various purposes such as providing insulation and reducing the energy expended on the maintenance of body temperature, protecting hatchlings from predators (nest predation and mortality is around 50–78%) (Lynch and Possidente, 1978; Willson and Gende, 2000; Thompson, 2007), and promoting parental interaction with the offspring during long rearing periods (Leighton, 2016). The nest may even serve as a catalyst for social behavior. It also shows information about the nest builder, such as physical strength, vigor, technical ability, foraging ability, or willingness to invest in the hatchlings later, or about the condition of its mate (Soler et al., 1998; Moreno, 2012). This information is important because females can modify their incubation and nestling-rearing responses according to male nest construction signals (Sheldon, 2000). Of note, not all bird species build nests, such as some sandpipers and plovers, but instead lay their eggs in shallow depressions in the ground, which can be lined with plants and feathers in some species (scrape-nesting) (Hansell, 2000).

Both males and females can be involved in nest building (Zuk and Bailey, 2008), but its construction implies severe energetic costs (Stanley, 2002; Antonov, 2004). A correct structure and composition of the nest can ensure success in the survival of the builders and their offspring (Altamirano et al., 2019) during breeding and non-breeding seasons (Hansell, 2000). Regarding the structure of the nest, the locations and preference for certain sites to build or form a nest depend on factors such as offspring protection and favor their growth. For example, weaver bird nests with hanging entrances to protect offspring from snakes (Healy, 2022), while the size and the weight of nests of robins, warblers, and finches increase to build at cooler temperatures in the northern versus southern latitudes (Crossman et al., 2011). Some birds build nests that can measure up to 1.5 m and be so resistant that they can support the weight of a human, such is the case of the African Hamerkop (Vergara et al., 2010). In terms of the processing material, Seitz and Zegers (1993) pointed out that this does influence the success of the nest, however, authors like Willson and Gende (2000), indicated that they did not find differences in different strata.

Additionally, DeGregorio et al. (2016) found that nest height (less than 1.1 m high) is more likely to be preyed but the type of predator changes according to the nest characteristics (high nests are preyed by other birds, while low nesting is associated with predation by snakes and rodents). Nest construction is also related to phenotypic quality in birds, reflecting the health status of the builder (Moreno, 2012). Therefore, the construction of the nest is not only for breeding purposes but also serves to give the mate some indicators to select the individual for mating.

### 2.2. Laying and incubation

Incubation, the next step after egg-laying, is a stage that affects the success of hatching, nestling growth, fledging, and post-fledging survival (Wang and Beissinger, 2011; Cones and Crowley, 2020). During this phase, females stay at the nest to provide protection and heat to the eggs, while the male forages for food. However, when optimal conditions are not met, events such as the so-called “partial incubation,” where the parents sit on the nest irregularly, are observed due to the parents leaving the nest for prolonged periods. This could affect the egg and nest temperature (Wang and Beissinger, 2011).

Offspring of precocial birds are characterized by leaving the nest almost immediately after hatching. This is relevant because it means that the incubation period and the changes during this stage directly affect the young. In the case of the Japanese quail (*Coturnix japonica*), an adaptative and protective behavior is present in which females decide the color of the nest according to the egg’s appearance. When laying heavily maculated eggs, the female selects a substrate that matches the color of the maculation, while laying lightly maculated eggs is seen in substrates with a light and similar color to that of the egg background (Lovell et al., 2013). Regarding environmental temperature, differences between temperate and tropical birds have been reported. For species in temperate environments (5–20°C), breed time extends for an average of 3–4 months, while tropical species (−5 to 40°C) require 7–8 months (Griffith et al., 2016). These differences have been associated with life traits such as a reduced metabolic rate in tropical birds and the small number of offspring in these birds due to the slow rate of growth after hatching (Wiersma et al., 2007). Also, the latitude is another factor that is related to the clutch size, where the higher the altitude the higher the clutch size. In tropical birds, mostly located at 2–6 km of altitude and nearest to the equator, their clutches are smaller and also require long-time to care for the young, have low growth rates, can have fewer resources during the year and nest predation is higher for tropical birds (McNamara et al., 2008; Londoño et al., 2015).

Similarly, the development rate of the hatchlings can be affected by environmental temperature. An example of this was studied by Hepp and Kennamer (2018) in wood ducks (*Aix sponsa*), in which temperature values of 35.8°C at incubation resulted in an appropriate development rate even in late-laid eggs. In contrast, incubation at 34.9° or 37.6°C resulted in a reduced number of eggs (183 and 160 vs. 222 at 35.8°C, respectively) and fewer nest (21 and 16 vs. 23). In the same species, DuRant et al. (2013) found that high temperatures at incubation (37.0°C) improve the thermoregulatory performance of ducklings after hatching. Therefore, the incubation period is not solely restricted to providing protection and conditions for the newborn to hatch, but also includes external factors.

### 2.3. Hatching

Data regarding environmental temperature are relevant due to climate change that could affect tropical species and their adaptation. High atmospheric temperatures also have consequences on hatching and offspring care. Studies demonstrated that high temperatures are associated with low growth rates of nestling due to the altered foraging behavior of parents (van de Ven et al., 2020). Likewise, during embryogenesis, daily exposure to 39°C during incubation retards organogenesis and affects chick performance in Dokki chickens (Abuoghaba et al., 2021). Additionally, locomotor behavior in bobwhite quail hatchlings is also altered when exposed to a temperature of 38.1°C during early incubation, delaying bone growth, reducing body mass, and altering the structure of intertarsal joints (Belnap et al., 2019). These temperature changes do not only influence the first post-hatching development but future survival and reproductive traits of birds (DuRant et al., 2013). To enhance reproductive success, females incubating eggs in several types of challenging environments can adjust the clutch size to reduce offspring mortality and their energy demands (Cones and Crowley, 2020).

Talking about environmental temperature, precocial hatchlings are born relatively independent, do not have many thermoregulatory constraints, and most of them can fly within a few days after hatching (Vleck and Vleck, 2011). In the case of broiler chickens, temperatures around 39.2°C improved hatchability and performance (Costa et al., 2020). Parent foraging for food is one the main reason why a temperature drop inside the nest is observed. Bertin et al. (2018) reported in domestic chicks (*Gallus gallus*) that *in ovo* exposure to a suboptimal temperature of 27.2°C delayed hatching and caused a higher prevalence of neophobia together with neuronal changes in the amygdala with a higher expression of corticotropin-releasing factor, an element associated to fear response. In contrast, Griffith et al. (2016) showed that a difference of +6°C within the nest reduced hatching time by approximately 3%. Therefore, although hatching is a stage that can be affected by several internal and external factors, birds rely different strategies to enhance offspring survival and performance, where attachment to the newborn is one of the first steps to providing care.

### 2.4. Filial imprinting

Newborn birds develop a preference for their parents (or a certain object) through a combination of visual, olfactory, auditory, and tactile cues (Bolhuis, 1991) in a process known as filial imprinting during the sensitive period (Bateson, 1966; Vallortigara and Versace, 2018). Through these sensory modalities, newborn birds can recognize their siblings and attach to their parents, enabling them to receive the necessary care and protection for survival (Vallortigara and Versace, 2018). For example, Bolhuis (1991) mentions that precocial animals such as ducklings and chicks use auditory stimuli to call their parents, while parent’s call form an attachment as a way to recognize other conspecifics.

The newborn attachment process begins before hatching and continues up to several weeks (Rogers, 1995). During this time, the chicks will imprint on their parents by recognizing the first moving object presented to them, as first shown by Lorenz (1937). This imprinting occurs during a sensitive period of short duration (first days of existence) and is irreversible. In turn, the parents will respond to the chicks’ vocalizations and behaviors. The parent birds will also protect the chicks from predators and teach them how to find food and survive in their environment. The chicks will also learn important skills such as flight, foraging, and socializing. Once the chicks have developed these skills, they will be ready to leave their parents and become independent.

The behavior of a hen toward her own brood or adopted chicks is different only on the first day (Richard-Yris, 1983). This seems to indicate that a mutual recognition exists between the mother and her young, nevertheless, innate reactions of adoption quickly occur. Alternatively, it was observed that chicks emit particular sounds when they are in the presence of food and that sound differs depending on the presence or absence of the mother (Nicol, 2004). Also, Collias (2000) indicates that the period spent with the mother is reassuring for the chicks and that the fearful and/or apprehensive behaviors only occur after the separation.

Interestingly, other studies with precocial young chicks (*Gallus gallus*) have found that early social interaction has genetic variability and is a trait that affects the ability of the chicks to establish social relationships during early life and when becoming adults (Versace et al., 2017, 2021; Lemaire and Vallortigara, 2023). Rosa-Salva et al. (2021) mention that although exposure to post-natal stimuli influences filial imprinting, social predispositions modify and direct the attention of the young birds, and this is a factor that can facilitate filial imprinting (Miura and Matsushima, 2016). For example, it is known that domestic chicks develop a strong preference to animate stimuli and face-like objects (or individuals) (Versace et al., 2016; Bliss et al., 2023; Lemaire and Vallortigara, 2023). This was reported in newly hatched chicks reared in darkness. In these animals, when exposed to a light sequence after the limited visual stimuli, the chicks had a predisposition to follow the patterns that were most similar to biological vertebrate motion regardless of the species (Vallortigara et al., 2005). Likewise, spontaneous preferences for faces and a bias of chicks to respond to an object with a structure similar to a face can be due to an innate conspecific detector pathway in birds (Rosa-Salva et al., 2009). Moreover, Versace and Vallortigara (2015) state that young behavior is the result of life experiences but social preferences even during early life involve unlearned knowledge or learning during the embryonic phase. Therefore, the social predisposition that precocial newborns seem to have modulates the learning and attachment responses at birth and during the sensitive period.

## 3. Parental behavior in altricial species

### 3.1. Nesting

As mentioned above, nest predation is one of the main causes of mortality in many bird species and particularly passerines (Cresswell, 1997). That is why many birds invest considerable time in the production and protection of their nests (Anderson et al., 1941), so it is vital to modify some behaviors such as reducing the clutch size (Lundberg, 1985) to improve survival rates. Ricklefs (1977) mentions that by modifying clutch size, parents ensure the number of offspring that can be nourished and protected, and that also might reduce nest predation by reducing feeding visits and the activity at the nest, making it less noticeable for predators. Presumably, a well-hidden nest could have an advantage over predators by reducing auditory, visual, and olfactory cues for potential predators (Mahesh and Lanka, 2022). However, not only is the nest well-concealed required but also that there is sufficient parental attendance to avoid or minimize any attack (Quader, 2006). To avoid being attacked by predators, birds must be alert while foraging (Thiruvenggadam et al., 2022), and this is why parental care provided by both females and males is very important in several species of birds such as white throated sparrows (*Zonoctrichia albicollis*), and house sparrows *(Passer domesticus).*

Like precocial species, altricial ones use different kinds of materials to elaborate their nests. To mention some examples, there are the ones made by Flamingos (*Phoenicopterus roseus*), which make mound nests by gathering mud to maintain heat inside the nest in cold conditions. Other types of nests are built on trees or any other open places and are called outstanding nests. Woodpeckers make cavities by excavating trunks and are called primary cavity-nesting birds. The birds that build nests in the abandoned nests or cavities of primary nesters are called secondary cavity-nesting birds, like House Sparrows (*Passer domesticus*), Grey Tits (*Parus major*), and Indian Robins (*Saxicoloides fulicata*) (Beardsell et al., 2016). Weavers (*Ploceidae*) form elaborate woven nests with different sizes, shapes, materials, and building techniques, depending on the species. For example, Baya weavers (*Ploceus philippinus*) build open habitats nest in farmlands, plantations, and paddy fields (Klatt et al., 2008; Mohring et al., 2021). The Rough-legged Hawk (*Buteo lagopus*) defines their nest site according to the number of small rodents found in the area and can reuse their nests for many consecutive years (Garson, 1980; Moreno et al., 2008). In the case of Buff-breasted wrens (*Thryothorus leucotis*), they build dormitory nests, which entails a large energy expenditure that puts their survival at risk (Berg et al., 2006).

They are also some nests that require a lot of energy in their elaboration, for example, the nests of female Pied Flycatchers (*Ficedula hypoleuca*) which are built mainly by females and can take 8–9 days (Moreno et al., 1995). Sometimes males build nests, such as in European Wrens (*Troglodytes troglodytes*), where the males build several nests and it is the females who select them according to the number of nests they have built and how resistant they are (Shields, 1984). This is how there can be two types of nests, such as those built by the Australian Reed Warblers (*Acrocephalus australis*), those that are for breeding and those that are for decoration to convince the females to reproduce with the male who built it (Chalfoun and Schmidt, 2012). Hence, the nest is a part of the courtship demonstration. The Chinstrap Penguin (*Pygoscelis antarctica*) make their nests with pebbles and the size of these nests is an indicator of the ability to defend the young for the mate, since other individuals in the colony tend to steal the pebbles to make their nests. Thus, stone collecting might reflect the building capacity of this species (Moreno, 2012; Zhao et al., 2016). Likewise, other nest-related behaviors such as longer travel times to the nest and offspring defense are part of the parental care of birds (Møller and Moller, 1990).

It is necessary to mention that the site where the bird chooses to make its nest is extremely important, not only so that predators cannot find it so easily, but also because reproductive success depends on it (Medlin and Risch, 2006; Quader, 2006). The selection of the sites where the nest is built is crucial, for example, blackbirds conceal nest sites to gain anti-predation benefits (Tómas et al., 2006). In a study carried out by Cresswell (1997), he evaluated the materials, clutch sizes, and characteristics of the nests, and he found that nests were built mostly in rhododendron, *Rhododendron* sp., bushes (47%, *n* = 145), or yew, *Taxus baccata*, trees or hedges (21%). And it was also found that nests were built significantly higher in yew trees (2.8 ± 0.4 m, *n* = 30), and lime, *Tilia* sp., trees (2.7 ± 0.8 m, *n* = 9) than in rhododendrons (1.6 ± 0.1 m, *n* = 59). Interestingly, some bird species (e.g., Great Crested Flycatchers (*Myiarchus crinitus*)) also utilize snake skins as nesting material to decrease predation by small mammalian predators such as squirrels (Martin, 1995).

Another important point to consider is the size of the nest. For example, female Blue tits (*Cyanistes caeruleus*) perform nest-building, and the size of it is associated with the health of the builder, since a large nest is representative of a phenotypic quality negatively associated with immunoglobulin levels and the presence of parasitic infections due to *Trypanosoma avium* (Herranz et al., 2004). There is controversy as to whether the size of the nest might or might not attract more or fewer predators. Some authors (Wesolowski, 2004; Biancucci and Martin, 2010; Kujala et al., 2022) pointed out that they can be striking, while others (Antonov, 2004), said that the size of the nest does not matter in this regard. This was observed by Kujala et al. (2022) in 22 altricial species of tropical birds, finding that nest predation was higher in larger nests and that predation might influence the selection of nest size.

### 3.2. Laying and incubation

Contrary to mammals, bi-parental care and cooperative breeding are common practices in avian species (Vleck and Vleck, 2011). Ninety percent of birds require this type of nursing where, generally, females incubate and brood while the male provides food (Schmaltz et al., 2008; Soler, 2017). Egg-laying in birds is mostly performed by singly laying (Schmaltz et al., 2008), such as the barn swallow (*Hirundo rustica*) which lays one egg per day and can incubate around 2–7 eggs (Saino et al., 2001). Differences may be present depending on the species (e.g., clutch size) and even on environmental factors (e.g., external temperature). For example, Wang et al. (2020) determined in the smooth-billed ani (*Crotophaga ani*) that females in larger groups produce more eggs and rely on competitive responses such as tossing or burying competitors’ eggs to improve their offspring survival while affecting the reproductive life of competitors.

According to Payne et al. (2000), females spend 60–83% of their time in the nest during the incubation stage, and males spend 35% of their time near the nest. Parental investment is expected to be higher in long-lived species that also have favorable environmental factors, low risk of predators, and good physical condition (Lynch et al., 2017). Regarding this aspect, a study carried out by Garson (1980) found that body condition at hatching in inexperienced females was significantly higher in more experienced birds. Besides, females reduce their incubation commitment when predation risk was high, independently of the hormone levels.

A particular laying behavior is observed in species that leave their eggs at the nest of other birds and do not provide parental care (Schmaltz et al., 2008), known as brood parasites (XirouchakKis and Mylonas, 2007). This kind of behavior has been studied in the common cuckoo (*Cuculus canorus*), where it was reported that egg-laying is performed during the whole day, except in the early morning to avoid host attack, and the time they spend in the host nest is around 2.56 and 26.28 s (Di Giovanni et al., 2022). Parasite species also adopt postures to avoid host-inflicted injuries by lowering their head and spreading their wings, as reported by Di Giovanni et al. (2022) in a study monitoring common cuckoos and oriental reed warblers (*Acrocephalus orientalis*). When considering the imprinting and offspring recognition in brood parasites, several studies have reported that young develop an imprinting-like behavior where traits such as mimicry songs of the host species (Pravosudov and Kitaysky, 2006) but can recognize and form affiliative bonds with conspecifics during adult life (Bowers et al., 2019). Alternatively, some of the parasitic chicks can actively kill their unrelated nestmates to take over all the food provided by the host parents, for which such parasitism represents supplementary energy costs by feeding extra mouths anyway (Vleck and Vleck, 2011).

### 3.3. Hatching

Several factors influence the incubation process and hatching success, such as the developmental stage according to altricial hatchlings. Altricial birds have asynchronous hatching, an event that might influence the appearance of a hierarchy inside the nest (Saino et al., 2001).

After hatching, altricial species require a constant food supply due to their rapid growth stage. The quantity and quality of food (especially the diet protein content through the provision of insect preys) each nestling gets influences its development, hence its survival (Vleck and Vleck, 2011). In the case of griffon vultures (*Gyps fulvus*), Martin (1993) reported that the parents continuously feed the nestling during the first 2 months after hatching, and 6–10 weeks old birds require the highest attention due to their fast-growing state during this time. Hatching failure in these species was studied by Lima and Dill (1990), who reported that according to non-predatory and embryonic mortality, failure rates ranged from 1 to 12.7% possibly due to poor incubation behavior or the inability of the parents to care and provide food to large clutches.

Nutritional stress is associated with the activation of the adrenocortical stress response and corticosterone release, a response that can be observed when *ad libitum* food is reduced by 60%, making corticosterone assessment in scrub jays (*Aphelocoma californica*) a predictor for fitness adequacy in adult life (Johnson, 1994). A way to assess the stress level and the outcome that this might represent is by quantifying the amounts of corticosteroids in the yolk. The levels of corticosterone in females during the nestling period (assessed by the presence of the steroid in yolks of freshly laid eggs) showed that females with high concentrations of corticosterone had a longer latency to resume parental care such as feeding and brooding the young. This is considered a self-maintenance survival priority over parental care (Evans and Stutchbury, 2012).

Besides, another factor that can affect the hatching success is the environmental temperature. For altricial birds (southern yellow-billed hornbill), the temperature must be maintained inside a narrow range according to the species (DuRant et al., 2013), usually between 35.5 and 38.5°C (Bertin et al., 2018). In the case of altricial hatchlings, they require constant brooding until they can thermoregulate by themselves. Relative humidity has also been reported as a factor that affects hatching, as seen in mourning doves (*Zenaida macroura*). In these doves, 90% of hatching success was reached when relative humidity values were between 35 and 45%, while only 50% of embryos hatched below a humidity of 95–100% (Walsberg and Schmidt, 1992).

### 3.4. Filial imprinting

It is known that many species of birds develop parental care for the young by the female and also by the male (Cockburn, 2006). By doing that, males can protect their paternity and increase mating success in taxa (Fedy and Martin, 2009). According to a study carried out by Payne et al. (2000), it was observed that in the species *Hylocichla mustelina*, the male and the female carry out coordinated care of the nest, where, when the female leaves the nest, the male is in charge of taking care of the broods, remaining at a distance no greater than 5 m from the nest to protect the hatchlings. The Scarlet Tanagers males (*Piranga olivacea*) (Berg et al., 2006), Red-faced Warbler (*Cardellina rubrifrons*), and Gray-headed Junco (*Junco hyemalis caniceps*) feed the females so that in this way they do not have to leave the nest in search of food and remain longer incubating the eggs (East, 1981). So, it is clear that guarding may provide an unrecognized form of indirect parental care by males.

Even if the young birds often leave the nest soon after hatching and are not dependent anymore on their parents for survival, some species of birds do form strong bonds with their offspring and provide significant care and protection for a longer time. For example, in some species of songbirds, such as the European Robin (*Erithacus rubecula*), the parents will continue to feed and protect their chicks for several weeks after they leave the nest. Storey et al. (2017) described that the young birds left the nest on average 12.8 day after hatching and they remained in their parents’ territories until independence. Fledglings became independent 19–20 days after leaving the nest. During the fledgling phase, young birds give “churring” calls when fed by parents and “contact” calls between feeds; these “contact” calls may not simply function as begging calls, they may also help to maintain contact between fledglings and parents.

So, filial imprinting is one of the most important behaviors in altricial species because they depend a lot on maternal care to secure their survival.

## 4. Neuroendocrine control of parental behavior in birds (precocial and altricial)

**Figure 2:**
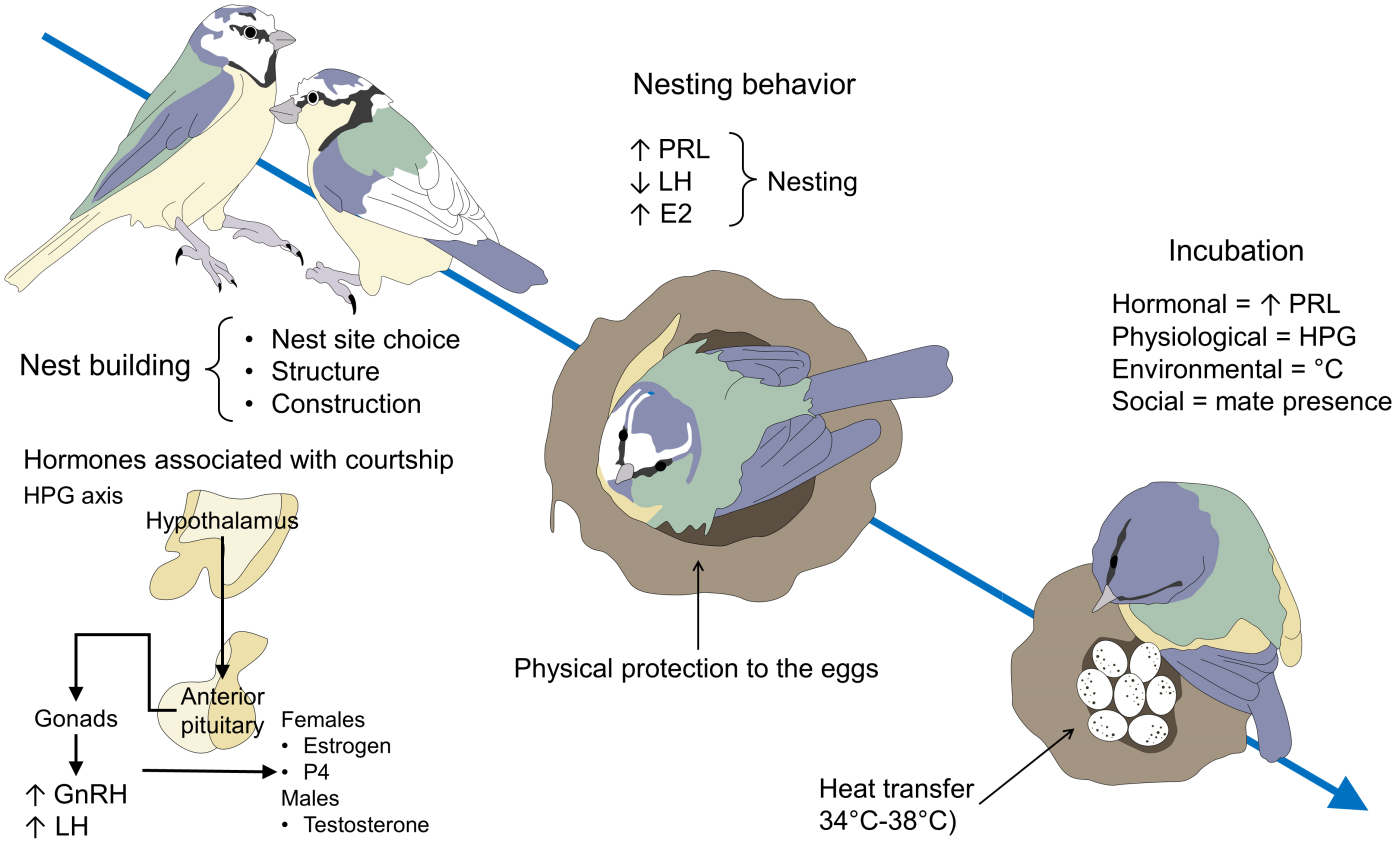
Neuroendocrine regulation of parental behavior in male and female birds. PRL is considered the main hormone involved in parental behavior in birds. However, its concentration levels differ depending on the reproductive stage. For example, in non-laying female birds, the interaction of VIP with D2R inhibits PRL release, contrary to what happens during incubation, where the interaction of VIP with D1R stimulates PRL release. The increase in PRL is maintained after hatching and gradually decreases as the breeding cycle progresses. Mesotocin, the homologous of mammalian oxytocin, has a similar pattern to PRL in females, peaking at incubation and rearing, particularly in brain areas such as the SON, POM, and PVN. In the case of testosterone, male birds tend to have the highest levels during the mating and non-breeding season and decrease when entering the parental phase. DA, dopamine; D1R, dopamine 1 receptor; D2R, dopamine 2 receptors; POM, medial preoptic area; PRL, prolactin; PVN, paraventricular nucleus; SON, supraoptic nucleus; VIP, vasoactive intestinal peptide.

Hormones play an important role in parental care and modulate the behavioral responses observed in each of the discussed stages. Figure 2 shows a general overview of the endocrine management of parental behavior in birds (Lovell et al., 2008; Chokchaloemwong et al., 2013; Kuenzel et al., 2015; Aleksandrova, 2019; Smiley, 2019; Kelly and Adkins-Regan, 2020).

### 4.1. Prolactin

When considering the endocrine control of laying, incubation onset, offspring defense, and provisioning, PRL is one of the most studied hormones in birds (Farrar et al., 2022) and is considered a hormone essential for incubation but not necessary after hatching (Vleck and Vleck, 2011; Ledwoń et al., 2022). Blood PRL concentrations are high during reproduction, and therefore, individuals that reach the highest blood PRL levels will be those who develop greater parental behavior, as well as higher brood provisioning (Garson, 1980; Smiley and Adkins-Regan, 2018a). PRL is hypothesized to inhibit reproductive hypothalamic–pituitary-gonadal (HPG) axis activity during parenting (Farrar, 2022). Thus, PRL can mediate crucial transitions from mating to parental behavior through a potential relationship with the gonadotropin-inhibitory hormone (GnIH) (Calisi et al., 2016). However, its effects on reproductive behaviors, such as courtship and copulation, need to be studied and may be species and breeding context-specific (Lea and Klandorf, 2002).

In females, nesting behavior is regulated by the interaction of estradiol, PRL, P4, vasoactive intestinal peptide (VIP), and follicle-stimulating hormone (FSH), whereas in males nest-building is highly influenced by female cues (González-Mariscal et al., 2005; Hall et al., 2015). In ring doves, Cheng and Silver (1975) determined that females receiving 50 and 100 μg of estradiol elicited nest-building and incubation. The same study reported that male behavior and nesting activity are not dependent on the same hormonal changes as the females, but males use female cues to incubate. This was also mentioned in male budgerigars (*Melopsittacus undulatus*) paired with estradiol-treated females. In these animals, paired males showed high courtship behavior in response to breeding females (Eda-Fujiwara et al., 2003).

For instance, in zebra finches (*Taeniopygia guttata*), endogenous PRL plays an important role during the laying stage and incubation by stimulating care toward filial and foster chicks, brooding (maintaining the chicks warm), and increasing the feeding rate by both parents (Smiley and Adkins-Regan, 2018b). In the same species, PRL levels increase gradually during incubation (from 4 ng/mL to approximately 11 ng/mL), peaking at hatching (around 14 ng/mL) and keeping an association with birds’ parental experience, where animals with prior experience have 50% higher PRL levels than animals without offspring (approximately 13 ng/mL vs. 8 ng/mL) (Smiley and Adkins-Regan, 2016). The same increase in PRL was found in rock doves (*Columba livia*) showing care for the offspring without affecting the reproductive function of the animals (Farrar et al., 2022). In the case of the zebra finch (*Taeniopygia guttata*), administration of a mesotocin receptor antagonist reduced nest building and incubation behaviors in females but not males, while an arginine-vasopressin (AVP) receptor antagonist reduced nest building in both sexes (Klatt and Goodson, 2013). Besides, experimental manipulations of the circulating levels of PRL (*via* injection of exogenous PRL or reduction of endogenous PRL) impact the incubation behavior, like in turkey hens (*Melleagris gallopavo*) (Youngren et al., 1991; El-Halawani et al., 2000). However, in the great tit (*Parus major*), no differences were found in feeding the young between males treated with testosterone and those who did not receive the drug (Van Duyse et al., 2002). In campo miner (*Geositta poeciloptera*), Lopes et al. (2021) found that testosterone levels declined near the laying date and kept decreasing with the beginning of parental care (from approximately 5 ng/mL to <2 ng/mL at 20 posture days), showing that males become less aggressive unless a territorial issue arises.

Triggering incubation requires coupling PRL with estradiol and P4 (Buntin, 1996). Plasma PRL concentrations increase at the onset of laying and stay elevated during a part or the entire parental phase, depending on the type of bird species (altricial vs. precocial). In most birds studied, PRL is high during incubation, although with different increase trajectories (Smiley, 2019). It has been observed that altricial birds (e.g., zebra finch) (Smiley and Adkins-Regan, 2018a) tend to show low levels of PRL during non-breeding times, then plasma PRL gradually increases at the laying phase and remains elevated after hatching, when the chicks need to be fed and guarded (Smiley, 2019). In the case of precocial birds, such as mallard ducks, Boos et al. (2007) studied the behavior and its association with PRL levels during the first 13 post-hatching days. The authors found that after 6 weeks, PRL levels decreased in parallel to a decline in parental care. After hatching, plasma PRL rapidly decreases in precocial birds, contrary to altricial individuals. It is the presence of altricial young but not parental feeding that stimulates PRL release in parent birds (Sharp et al., 1998). Moreover, experimental manipulations of PRL levels showed that low concentrations of PRL in zebra finches (an altricial species) reduce parental care in a study where PRL release was inhibited with bromocriptine 3 days before hatching and 2 days after hatching. The inhibition of PRL eliminated or drastically reduced chick brooding (from 83.3 to 30.8%) and feeding (from 66.7 to 20.8%), a factor that affected the nest temperature on post-hatching day one, recording lower temperatures than control groups (*p* = 0.05) (Smiley and Adkins-Regan, 2018a). In addition, Schoech et al. (1996) demonstrated in the Florida scrub jay (*Aphelocoma c. coerulescens*) that PRL concentrations were significantly correlated with the number of visits to the nest, as well as the amount of food delivered to the young.

### 4.2. Steroids

After clutch completion (the total number of eggs a bird lays per each nesting attempt), gonadotropins (LH, FSH) and gonadal steroids (testosterone, estradiol, progesterone) tend to decrease and remain low throughout the incubation and post-hatching phases in males and females (Lea and Klandorf, 2002).

**Figure 3:**
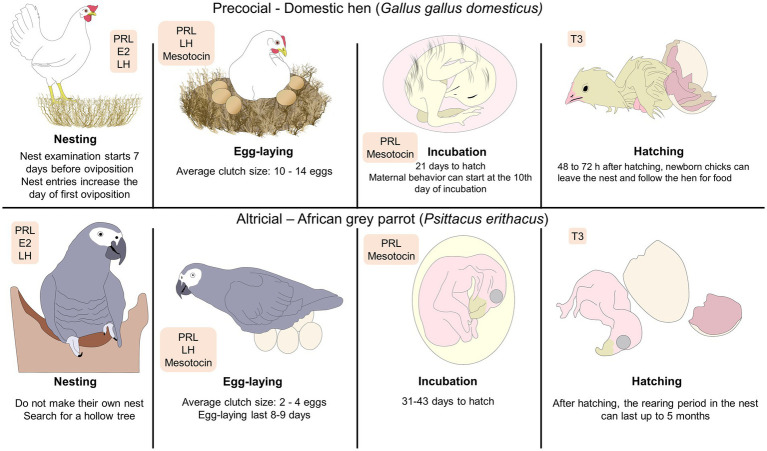
Hormonal influence on nest building and the first stages of egg-laying. Nest building, nesting behavior, and incubation of the eggs follow specific endocrine changes in all avian species. During nest building, the activation of the HPG axis and the consequent production of GnRH, LH, estrogen, P4, and testosterone participate to initiate this stage in both male and female individuals. Estradiol is particularly important for female courtship, nest-building, and the onset of incubation. For nesting and incubation, the main hormone involved is PRL, which concentrations increase to promote parental behavior before and after hatching. E2, estradiol; GnRH, gonadotropin-releasing hormone; HPG, hypothalamic–pituitary-gonadal; LH, luteinizing hormone; P4, progesterone, PRL, prolactin.

So, PRL promotes parental care and commitment, while corticosterone can promote or reduce parental investment (Garson, 1980). Figure 3 exemplifies the hormonal control of nest-building, nesting, and incubation in avian species. Corticosterone is also an important hormone involved in attachment and parental behavior. Both corticosterone and PRL increase during parental care because of their higher metabolic demands (Lowther and Johnston, 2020). Corticosterone is the hormone involved in presenting an adequate response to changes in environmental conditions and it has been observed that high levels of corticosterone in the blood can cause a decrease in parental care (Schoenle et al., 2017). Thus, PRL and corticosterone may balance the bird’s trade-offs between parental care efforts and their own survival. Additionally, parental experience may affect the corticosterone and PRL responses to challenges (Farrar et al., 2022). For instance, studies in the oldest seabirds show that the more aged individuals show lower stress-induced corticosterone and higher stress-induced PRL (Heidinger et al., 2010). Experienced birds alter hippocampal glucocorticoid (Farrar et al., 2022) and hypothalamic PRL receptors (Farrar et al., 2022), therefore rearing chicks can be a similar experience to those seen early in development, where the responsiveness of the HPG axis is altered later in life (Farrar et al., 2022).

### 4.3. Luteinizing hormone

Similarly, LH is involved in the different stages linked to reproduction/nesting/clutching/laying. In canvasback ducks (*Aythya valisineria*), increases in serum LH were reported in breeding females, pre-laying ducks, and during the laying period (Bluhm et al., 1983b). Also, high LH concentrations participate and stimulate the onset of nesting activity (Bluhm et al., 1983b). In precocial birds, plasma PRL levels are high immediately after egg laying, stay moderately elevated during the brooding period, and rapidly decrease after chick hatching (Angelier and Chastel, 2009; Ohkubo, 2017; Smiley and Adkins-Regan, 2018a).

### 4.4. Mesotocin

Another important nonapeptide in birds is the homolog of oxytocin: mesotocin, a hormone that is involved in behavior from incubation to brooding (Thayananuphat et al., 2011), as has been reported in chickens and turkeys (Kelly and Adkins-Regan, 2020). Both neurohypophysial hormones –blood mesotocin and arginine vasotocin– increase, inducing oviposition and uterine contractions in birds (Takahashi and Kawashima, 2008). In turkeys, high concentrations of mesotocin were found in the paraventriculus nucleus (PVN) and the nucleus supraopticus pars ventralis during incubation, a response that is similar to the reported in mammals during parturition and lactation (Thayananuphat et al., 2011). There is a study where mesotocin antagonist infusions have been administered to turkeys (*Melagris gallopavo*) and it has been seen that the behaviors of brooding chicks were considerably reduced (Thayananuphat et al., 2011). In the same way, there is one study made in chickens (*Gallus domesticus*) where it has been observed that the presence of mesotocin favors behaviors of nest attendance and care of the chicks (Chokchaloemwong et al., 2013).

Regarding mesotocin, its mRNA expression was found higher in the PVN of hens rearing chicks than in laying hens as early as just after the hatching, suggesting the involvement of mesotocin in rearing in the chicken (Aleksandrova et al., 2016). Additionally, the number of mesotocin-immunoreactive hypothalamic neurons (within the nucleus supraopticus, pars ventralis (SOv), nucleus preopticus medialis (POM), and nucleus paraventricularis magnocellularis) was low in non-laying native Thai hens, but it increased gradually when the hens entered the laying stage and peaked in incubating and rearing hens (Chokchaloemwong et al., 2013). Also, it increased in native Thai hens whose eggs had been replaced by newly hatched chicks as soon as 3 days after the substitution (Sinpru et al., 2018). In the case of native domestic chicks, the administration of bilateral intracranial mesotocin was associated with a high preference of the animals to initiate affiliative behaviors toward a stuffed hen after hatching, showing that this nonapeptide has a relevant role for social recognition immediately after birth (Loveland et al., 2019). These results emphasize the role of mesotocin in young rearing after hatching.

### 4.5. Other neuroendocrinological factors

On the other hand, during filial imprinting, authors such as Yamaguchi et al. (2012) reported that the thyroid hormone influences this process by modulating the sensitive period. In chicks, T3 initiates the sensitive period and also participates in the learning process by establishing preferences for certain objects or for their parents (Lorenzi et al., 2021). Moreover, protein synthesis and its activation (e.g., mammalian target of rapamycin complex 1) is another important element that has not been extensively studied but is involved in long-term memory formation that is necessary during filial imprinting (Batista et al., 2018).

Due to all of the above, it can be said that both in altricial and precocial species, the main hormones involved in the development of parental care for the offspring are PRL, P4, mesotocin, LH, and FSH.

## 5. Conclusion

In this review, we attempted to provide a general overview of the birds’ parental and filial behavior and endocrinology, examining the different strategies employed on one hand, by precocial species and on another hand, by altricial species, as well as the various factors that intervene in the establishment of parental behavior and newborn attachment, such as life history and hormonal influence. Dealing with such a broad topic implies the following limitation: we could not get into detailed and specific information regarding every species of birds.

Unlike some species, in birds, parental care can be done by the female, the male, or both, which helps improve hatchling survival rates.

Various factors intervene in the survival of the young birds and parental attachment; among them, the development of an adequate nest (size, material, site, purpose), a good physical condition of the parents, the commitment of the female in the incubation and of the male in coordination with the female to provide parental care, the correct release of hormones that are triggered during these processes, parental experience and the incidence of predatory situations. It has been seen that another important aspect is the filial imprinting which means that both the mother and the offspring can be recognized either by visual or auditory stimuli and in this way, along with the approach to the imprinted stimulus, the survival of the newborns is favored through maternal care. In hatching, other factors can be involved, like the environmental temperature, whether the species are altricial or precocial, and nutritional stress.

## Author contributions

All authors contributed to the conceptualization, writing, reading, and approval of the final manuscript.

## Conflict of interest

The authors declare that the research was conducted in the absence of any commercial or financial relationships that could be construed as a potential conflict of interest.

## Publisher’s note

All claims expressed in this article are solely those of the authors and do not necessarily represent those of their affiliated organizations, or those of the publisher, the editors and the reviewers. Any product that may be evaluated in this article, or claim that may be made by its manufacturer, is not guaranteed or endorsed by the publisher.
